# The first postoperative-stimulated serum thyroglobulin is a prognostic factor for thyroid microcarcinomas^☆^

**DOI:** 10.1016/j.bjorl.2017.10.005

**Published:** 2017-10-31

**Authors:** Isabela de Oliveira Amui, José Vicente Tagliarini, Emanuel C. Castilho, Mariângela de Alencar Marques, Yoshio Kiy, José Eduardo Corrente, Gláucia M.F.S. Mazeto

**Affiliations:** a Universidade Estadual Paulista “Júlio de Mesquita Filho” (Unesp), Faculdade de Medicina de Botucatu, Departamento de Medicina Interna, Botucatu, SP, Brazil; b Universidade Estadual Paulista “Júlio de Mesquita Filho” (Unesp), Faculdade de Medicina de Botucatu, Departamento de Oftalmologia, Otorrinolaringologia e Cirurgia de Cabeça e Pescoço, Botucatu, SP, Brazil; c Universidade Estadual Paulista “Júlio de Mesquita Filho” (Unesp), Faculdade de Medicina de Botucatu, Departamento de Patologia, Botucatu, SP, Brazil; d Universidade Estadual Paulista “Júlio de Mesquita Filho” (Unesp), Faculdade de Medicina de Botucatu, Departamento de Doenças Tropicais e Diagnóstico por Imagem, Botucatu, SP, Brazil; e Universidade Estadual Paulista “Júlio de Mesquita Filho” (Unesp), Instituto de Biociências, Departamento de Bioestatística, Botucatu, SP, Brazil

**Keywords:** Biological markers, Clinical evolution, Prognosis, Thyroglobulin, Thyroid neoplasms, Marcadores biológicos, Evolução clínica, Prognóstico, Tireoglobulina, Neoplasias da tireoide

## Abstract

**Introduction:**

Endogenous thyroid-stimulating hormone-stimulated thyroglobulin collected after total thyroidectomy is a useful predictor of better prognosis in patients with differentiated thyroid carcinomas in general, but studies with microcarcinomas are scarce.

**Objective:**

To assess whether the first postoperative stimulated thyroglobulin measurement is a prognostic factor in patients with microcarcinoma.

**Methods:**

The medical data of 150 differentiated thyroid carcinoma patients were studied retrospectively, and 54 (36%) cases with microcarcinoma were selected. The first postoperative stimulated thyroglobulin (1st stimulated thyroglobulin), measured after thyroidectomy, initial presentation data, and microcarcinomas treatment were assessed regarding outcome. Worse prognosis was defined as neoplasm persistence/recurrence.

**Results:**

Persistence/recurrence occurred in 27.8% of the cases. These patients were identified according to the following parameters: receiving more than one ^131^iodine dose (100% vs. 0%; *p* < 0.0001); accumulated ^131^iodine dose (232.14 ± 99.09 vs. 144 ± 33.61 mCi; *p* < 0.0001); presented active disease in the last assessment (53.3% vs. 0%; *p* < 0.0001); follow-up time (103.07 ± 61.27 vs. 66.85 ± 70.14 months; *p* = 0.019); and 1st stimulated thyroglobulin (19.01 ± 44.18 vs. 2.19 ± 2.54 ng/dL; *p* < 0.0001). After multivariate logistic regression, only the 1stSTg [odds ratio = 1.242; 95% confidence interval: 1.022–1.509; *p* = 0.029] and follow-up time (odds ratio = 1.027; 95% confidence interval: 1.007–1.048; *p* = 0.007) were independent predictors of risk of persistence/recurrence. The cutoff point of 1.6 ng/dL for the 1st stimulated thyroglobulin was significantly associated with disease persistence/recurrence [area under the curve = 0.713 (*p* = 0.019)].

**Conclusion:**

The first stimulated thyroglobulin predicted disease persistence/recurrence in patients with microcarcinoma.

## Resumo

**Introdução:**

A tireoglobulina estimulada pelo hormônio tireoestimulante endógeno coletada após tireoidectomia total é um preditor útil de melhor prognóstico em pacientes com carcinomas diferenciados de tireoide em geral, mas os estudos com microcarcinomas são escassos.

**Objetivo:**

Avaliar se a primeira medida pós-operatória de tireoglobulina estimulada é um fator prognóstico em pacientes com microcarcinoma.

**Método:**

Os dados clínicos de 150 pacientes com carcinoma diferenciado de tireoide foram estudados retrospectivamente e 54 (36%) casos com microcarcinoma foram selecionados. A primeira dosagem de tireoglobulina estimulada (1^a^TgE) pós-operatória, medida após a tireoidectomia, os dados da apresentação inicial e tratamento do microcarcinoma foram avaliados quanto ao resultado. O pior prognóstico foi definido como a persistência/recorrência da neoplasia.

**Resultados:**

A persistência/recorrência ocorreu em 27,8% dos casos. Esses pacientes foram identificados de acordo com os seguintes parâmetros: receberam mais de uma dose de iodo^131^ (100% vs. 0%; *p* < 0,0001); dose acumulada de iodo^131^ (232,14 ± 99,09 vs. 144 ± 33,61 mCi; *p* < 0,0001); apresentou doença ativa na última avaliação (53,3% vs. 0%; *p* < 0,0001); tempo de seguimento (103,07 ± 61,27 vs. 66,85 ± 70,14 meses; *p* = 0,019); e 1ªTgE (19,01 ± 44,18 vs. 2,19 ± 2,54 ng/dL; *p* < 0,0001). Após a regressão logística multivariada, apenas a 1ªTgE [*odds ratio* = 1.242; intervalo de confiança de 95%: 1,022-1,509; *p* = 0,029] e tempo de seguimento (*odds ratio* = 1,027; intervalo de confiança de 95%: 1,007-1,048; *p* = 0,007) foram preditores independentes de risco de persistência/recorrência. O ponto de corte de 1,6 ng/dL para a 1ª TgE foi significativamente associado à persistência/recidiva da doença [área abaixo da curva = 0,713 (*p* = 0,019)].

**Conclusão:**

A 1ª dosagem sérica de tireoglobulina estimulada previu a persistência/recorrência da doença em pacientes com microcarcinoma.

## Introduction

The incidence of differentiated thyroid carcinoma (DTCs) has been growing significantly,^1^ especially because of higher microcarcinoma (TMC) frequency.^2^, ^3^ Although TMC are generally associated with excellent prognosis,^4^ some patients have more aggressive tumors, resulting in higher rates of persistency/recurrence and active disease in the long-term follow-up.^5^ Thus, many TMC-related clinical, histopathological, and molecular parameters with varying complexities and costs have been assessed in the search for markers that can predict higher aggressiveness and worse prognosis.^6^ Nevertheless, these parameters vary from one study to another, and the factors associated with worse prognosis have not yet been completely established, preventing consensus on the most effective TMC treatment approach. Larger tumors, multifocality, and capsular invasion have been associated with lymph node metastasis,^7^ while younger age, multifocality, subcapsular location, extrathyroidal extension, intraglandular tumor fibrosis, and BRAF mutation have been associated with higher recurrence.^8^, ^9^

In this context, a single serum thyroid-stimulating hormone (TSH)-stimulated thyroglobulin (STg) measurement after total thyroidectomy has been useful for predicting a better prognosis in DTC patients.^10^ Yet studies that assess this parameter specifically in patients with TMC are scarce. This study assessed whether the first postoperative STg measurement is a prognostic factor in TMC patients.

## Methods

This retrospective study assessed the clinical course of TMC patients and compared the first postoperative STg (1stSTg), and many other clinical, laboratory, and therapeutic parameters of patients with and without tumor persistence/recurrence after initial treatment. This study was approved by the Research Ethics Committee of the institution in which it was conducted (protocol n° 4288-2012).

### Patients

The medical data of 150 late postoperative DTC patients were assessed. The patients were being followed in an outpatient clinic of thyroid neoplasms of a tertiary hospital in Brazil. Fifty-four (36%) TMC patients submitted to total thyroidectomy (TT) between 1994 and 2010 were selected. These patients did not have other thyroid neoplasms, were not positive for antithyroglobulin antibodies (TgAb), had postoperative follow-up of at least 24 months, and were taking levothyroxine.

The service's treatment/follow-up protocol of DTC patients at the time the cases were enrolled in the study consisted of TT, followed by diagnostic whole-body scan (WBS), and serum endogenous TSH-stimulated thyroglobulin (1stSTg) measurement three months after TT. The patients then received an ablative/therapeutic dose of radioactive iodine (TDI) followed by confirmatory WBS 5 days later. One year after TDI, STg and TSH were measured, and a neck ultrasound (US) was performed. Clinical and laboratory assessments were performed each 4 or 6 months, which included dosing of serum TSH, free thyroxine (FT4), TgAb, and thyroglobulin (Tg). Neck US and chest X-ray were performed annually, and other imaging tests [chest computed tomography (CT), abdominal US, neck and mediastinal magnetic resonance imaging (MRI), new WBS and positron emission tomography (PET-CT)] or cytohistological tests were requested upon suspicion of active disease.

TMCs were defined as tumors observed in the histopathological analysis with largest diameter of 1.0 cm or smaller and histological diagnosis of papillary carcinoma (PC), follicular carcinoma (FC), or Hürthle cell carcinoma.^11^

### Study parameters

The main variable of interest was the 1stSTg. Nevertheless, the general characteristics of the patients, initial presentation of the neoplasm, treatment, and disease outcome were also assessed. Cases with and without disease persistence/recurrence were compared with regards to these parameters to determine possible predictors of the outcome persistence/recurrence. Patients were initially characterized by gender, age at the time of surgery, self-reported race, and initial disease presentation, which considered the following: tumor characteristics and stage [risk of recurrence (LATS) and mortality (TNM)],^12^, ^13^ first postoperative WBS (WBS was considered positive if any uptake in any segment was detected by scintigraphy), and percentage of ^131^Iodine (^131^I) uptake. Treatment-related aspects were also assessed, such as neck dissection during TT, number of ^131^I doses, and total accumulated dose (in mCi).

Disease outcome was assessed mainly according to tumor persistence or recurrence. The following were also evaluated: patient's condition in the last assessment, whether with or without active disease; disease-free survival time (in months); and follow-up time (in months). Disease persistence or recurrence was defined as STg ≥ 2 ng/mL, or active disease evidenced by imaging tests or biopsy one year after the initial treatment (TT and WBS).^14^, ^15^ Active tumor in the last assessment was defined as death caused by the tumor or presence of the same criteria used for defining persistence or recurrence.

FT4, TSH, and Tg were determined by chemiluminescence (DPC, Los Angeles, CA, USA) at the clinical laboratory of Hospital das Clínicas – Faculdade de Medicina de Botucatu. The reference values for FT4 and TSH were 0.80–1.90 ng/dL and 0.40–4.0 μIU/mL, respectively, while those for Tg were 0.83–68.0 ng/mL. Tg analytical and functional sensitivities were 0.2 ng/mL and 0.9 ng/mL (for values higher than 2 ng/mL), respectively.

### Statistical analyses

The variables underwent univariate analysis in relation to tumor persistence or recurrence. Only age had symmetric distribution, so it was assessed by the Student's *t*-test. The other numerical variables (means ± standard deviations, SD) were adjusted by the generalized linear model with a gamma distribution (asymmetric). The qualitative variables (percentages) were assessed by the Fisher's exact test. Later, multivariate logistic regression was performed with the univariate analysis variables with *p* ≤ 0.15. The response variable was tumor persistence or recurrence. The variables were selected by the stepwise method.

A receiver-operating characteristics (ROC) curve was constructed for the 1stSTg to establish the cutoff and determine the marker's sensitivity and specificity to predict tumor persistence or recurrence. The significance level was set at 5% (*p* < 0.05).

## Results

Table 1 shows the patients’ general data. Five patients (9.3%) had recurrence and 15 (27.8%) had persistence/recurrence, of which 8 (53.3%) still presented active disease in the last medical assessment. Distant metastases or deaths during the follow-up period did not occur.

**Table 1:** Clinical and histopathological data of patients.

General data	
*Female, n (%)*^a^	48 (88.9)
*White reported color, n (%)*^a^	53 (98.2)
*Age (years)*^b^	46.30 ± 13.58
*Follow-up (months)*^b^	76.91 ± 69.19

*Total thyroidectomy, n (%)*^a^	
One stage	33 (61.1)
Two stages	21 (38.9)

*Lymph node dissection, n (%)*^a^	16 (29.6)
*Histological subtypes, n (%)*^a^	
*Papillary carcinoma*	
Classic	41 (75.9)
Follicular variant	8 (14.8)
Sclerosing	1 (1.8)
Mucinous	1 (1.8)
Columnar cells	1 (1.8)
Oncocytic cells	1 (1.8)
Follicular	1 (1.8)

*Tumor size (cm)*^b^	0.61 ± 0.30
*Multifocality, n (%)*^a^	20 (37.0)
*Bilaterality, n (%)*^b^	15 (27.8)

*Tumor capsule, n (%)*^a^	
Complete	13 (24.1)
Incomplete	8 (14.8)
Absent	33 (61.1)
Lymph node metastases, *n* (%)^a^	7 (13)

*TNM staging, n (%)*^a^	
I	44 (81.5)
III	1 (1.8)
IV	9 (16.7)

*1st whole body scan positive, n (%)*^a^	51 (94.4)
*1st Thyroglobulin stimulated (ng/dL)*^b^	6.72 ± 23.6

*Number of doses of 131 Iodine, n (%)*^a^	
0	1 (1.9)
1	44 (81.5)
2	8 (14.8)
3	1 (1.9)

*Iodine uptake (%)*^b^	1.51 ± 1.65
*Cumulative dose of 131 Iodine (mCi)*^b^	167.79 ± 69.84
*Recurrence, n (%)*^a^	5 (9.3)
*Persistence/recurrence, n (%)*^a^	15 (27.8)
*Active disease in the last medical evaluation, n (%)*^a^	8 (14.8)
*Disease-free survival (months)*^b^	42.06 ± 65.03

cm, centimeters; mCi, milicuries; *n*, number; ng/dL, nanograms per decilitre; %, percentage; TNM, tumor-node-metastases, staging system of the American Joint Commission on Cancer (AJCC).^13^

a Frequencies and percentages for categorical variables.

b Mean ± standard deviation.

The group with disease persistence/recurrence had higher 1stSTg level (*p* < 0.0001), accumulated ^131^iodine dose (*p* < 0.0001), follow-up time (*p* = 0.019), percentage of patients who received two or more ^131^I doses (*p* < 0.0001), and percentage of patients with active disease in the last assessment (*p* < 0.0001) (Table 2).

**Table 2:** Comparative analysis^a^ of clinical and histopathological data between patients with and without cancer persistence/recurrence.

General data	Persistence/recurrence of the disease		*p*
	No *n* = 39 (72.2%)	Yes *n* = 15 (27.8%)	
Age (years)	44.87 ± 13.19	50.00 ± 14.32	0.217
Female, *n* (%)	36 (92.3)	12 (80.0)	0.197
Total thyroidectomy in two stages, *n* (%)	15 (38.5)	6 (40.0)	0.917
Lymph node dissection, *n* (%)	10 (25.6)	6 (40.0)	0.301
Tumor size (cm)	0.63 ± 0.29	0.57 ± 0.33	0.618
Multifocality, *n* (%)	15 (38.5)	5 (33.3)	0.727
Bilaterality, *n* (%)	11 (28.2)	4 (26.7)	0.946
Classic papillary carcinoma, *n* (%)	31 (79.5)	10 (66.7)	0.324
Encapsulated tumor, *n* (%)	8 (20.5)	5 (33.3)	0.324
Invasion of tumor capsule, *n* (%)	4 (10.3)	3 (20.0)	0.306
Lymph node metastases, *n* (%)	5 (12.8)	2 (13.3)	0.960
Contralateral lymph node metastases, *n* (%)	2 (5.1)	2 (13.3)	0.147
TNM III/IV, *n* (%)	8 (20.5)	2 (13.3)	0.543
1st thyroglobulin stimulated (ng/dL)	2.19 ± 2.54	19.01 ± 44.18	<0.0001
^131^Iodine uptake (%)	1.57 ± 1.65	1.36 ± 1.45	0.687
1st whole body scan positive, *n* (%)	36 (92.3)	15 (100.0)	0.269
Cumulative dose of ^131^Iodine (mCi)	144.08 ± 33.61	232.14 ± 99.09	<0.0001
Follow-up (months)	66.85 ± 70.14	103.07 ± 61.27	0.019
Two or more doses of ^131^Iodine, *n* (%)	0 (0.0)	9 (60.0)	<0.0001
Disease-free survival (months)	39.44 ± 69.56	48.87 ± 52.97	0.116
Active disease in the last evaluation, *n* (%)	0 (0.0)	8 (53.3)	<0.0001

cm, centimeters; mCi, milicuries; *n*, number; ng/dL, nanograms per decilitre; %, percentage.

a Univariate analysis of categorical variables (*n* and %; Fisher's exact test) and numerical [mean ± standard deviation; Student's *t* test for age and adjustment for generalized linear model with gamma distribution (asymmetrically), for the other variables] for the presence of persistence and/or recurrence of cancer. Significance: *p* < 0.05. The variables with *p* ≤ 0.15 in the univariate analysis were evaluated subsequently by the multivariate analysis.

In multivariate logistic regression, 1stSTg [odds ratio (OR) = 1.242; 95% confidence interval (CI): 1.022–1.509; *p* = 0.029] and follow-up time (OR = 1.027; 95% CI: 1.007–1.048; *p* = 0.007) were independent predictors of risk of DTC persistence/recurrence.

**Figure 1:**
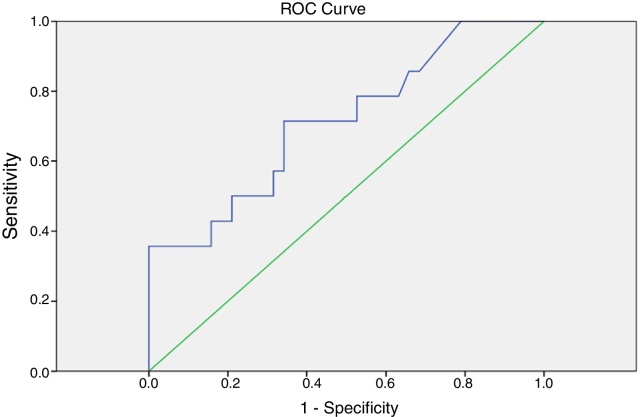
Receiver-operating characteristic curve (ROC) of the first stimulated thyroglobulin [cutoff = 1.6 ng/dL (area under the curve: 0.713; *p* = 0.019)] as predictor of cancer persistence/recurrence.

**Figure 2:**
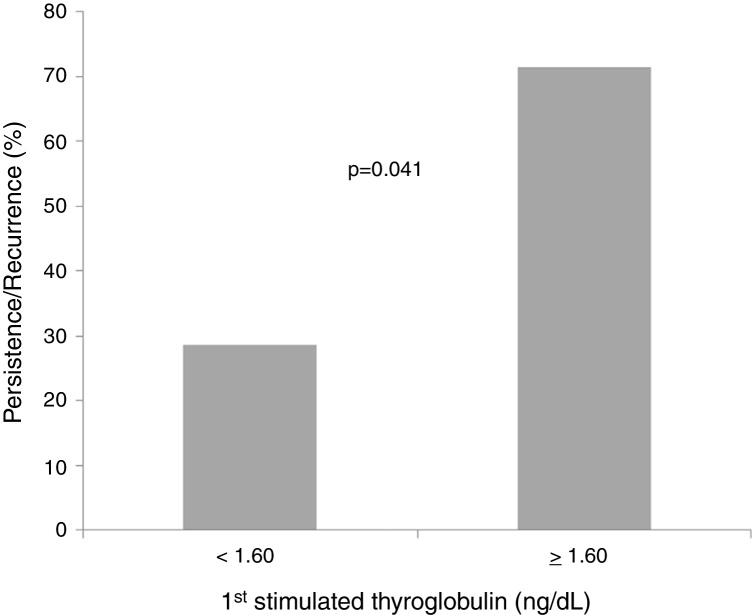
Persistence/recurrence of the tumor in relation to the first stimulated thyroglobulin (smaller or greater than 1.60 ng/dL). Chi-square test. Significance: *p* < 0.05.

Based on the ROC curve, the 1stSTg cutoff of 1.6 ng/dL was associated with a sensitivity of 70% and a specificity of 60% (area under the curve = 0.713; *p* = 0.019) for tumor persistence/recurrence (Fig. 1). Most patients (71.4%) with 1stSTg level equal to or greater than 1.6 ng/dL had tumor persistence/recurrence, and most cases (60.5%) with STg level below 1.6 ng/dL did not (Fig. 2).

## Discussion

Serum STg determination after TT and before ^131^I ablation, herein called 1stSTg, could help to predict the initial response to therapy and DTC prognosis.^10^, ^16^, ^17^ However, most studies assess DTC in general and do not investigate the 1stSTg specifically in patients with TMC. This study found that 1stSTg can be an independent predictor of carcinoma persistence/recurrence also for these tumors. This marker remained significant even when assessed together with other parameters frequently associated with TMC prognosis.^8^, ^9^, ^18^, ^19^, ^20^

An important topic of discussion is the optimal 1stSTg cutoff for the prognosis. For DTCs in general, levels between 20 and 30 ng/mL have been associated with higher sensitivity and specificity for predicting disease persistence/recurrence, while levels <1–2 ng/mL would be strong predictors of remission.^4^ In a recent meta-analysis with almost 4000 patients, Webb et al. found high negative predictive value for disease-free status when pre-ablation serum Tg was below 10 ng/mL.^10^ However, the exact Tg levels required to prognosticate DTCs in general or TMCs have not been established as they depend on many factors, such as TSH level,^16^ assay sensitivity, and amount of residual tissue, among others.^4^ The cutoff found by the present study for TMC (1.6 ng/dL) was much lower than the cutoffs mentioned earlier, with 70% sensitivity and 60% specificity to predict disease persistence/recurrence. This finding may be explained by many reasons. First, considering that all the study patients underwent TT, and the ^131^I uptake after surgery and before ablation was relatively low, we infer that the remaining cervical tissue must have been scanty, which could at least partly explain the lower cutoffs. Moreover, since Tg tends to reach its nadir around three to four weeks after TT,^4^ it could have continued to decrease after this initial period.^15^ Hence, since we assessed STg about three months after surgery, this longer interval could have contributed to the lower cutoffs.

Although the rate of TMC recurrence is not high, especially in patients submitted to TT,^21^ it is not negligible. The study rates of disease persistence/recurrence and active disease in the last assessment were almost 30% and 15%, respectively. Therefore, we believe that the therapeutic approach should be individualized, and that STg could be one of the parameters included in this individualization. Based on this study results, in patients with negative TgAb, a STg level below 2 ng/dL, measured in the first three months after TT and before eventual therapeutic ^131^I dose, indicates good prognosis in TMC patients.

The limitations of this study could have influenced the results and include: its retrospective character, the modest sample size, the various histologic subtypes included (some of them with worse prognosis), the inability to classify the cases according to initial disease presentation (incidental or non-incidental TMC),^22^, ^23^ and the initial treatment of the patients (total thyroidectomy and therapeutic dose of ^131^I), which has not been currently indicated for TMC.^4^ Nevertheless, this study's merit is bringing to light the importance of measuring STg after thyroidectomy to prognosticate TMC.

## Conclusion

The first postoperative STg measurement was capable of predicting TMC persistence/recurrence. Other studies with larger sample sizes and different designs are necessary to confirm these results.

## Conflicts of interest

The authors declare no conflicts of interest.

## References

[1] Veiga L.H., Neta G., Aschebrook-Kilfoy B., Ron E., Devesa S.S. (2013). Thyroid cancer incidence patterns in São Paulo, Brazil, and the U.S. SEER program, 1997–2008. Thyroid.

[2] Cordioli M.I., Canalli M.H., Coral M.H. (2009). Increase incidence of thyroid cancer in Florianopolis, Brazil: comparative study of diagnosed cases in 2000 and 2005. Arq Bras Endocrinol Metabol.

[3] Yu X.M., Wan Y., Sippel R.S., Chen H. (2011). Should all papillary thyroid microcarcinomas be aggressively treated? An analysis of 18,445 cases. Ann Surg.

[4] Haugen B.R., Alexander E.K., Bible K.C., Doherty G.M., Mandel S.J., Nikiforov Y.E. (2016). 2015 American Thyroid Association Management Guidelines for adult patients with thyroid nodules and differentiated thyroid cancer: the American thyroid association guidelines task force on thyroid nodules and differentiated thyroid cancer. Thyroid.

[5] Friguglietti C.U., Dutenhefner S.E., Brandão L.G., Kulcsar M.A. (2011). Classification of papillary thyroid microcarcinoma according to size and fine-needle aspiration cytology: behavior and therapeutic implications. Head Neck.

[6] Grodski S., Delbridge L. (2009). An update on papillary microcarcinoma. Curr Opin Oncol.

[7] Vasileiadis I., Karakostas E., Charitoudis G., Stavrianaki A., Kapetanakis S., Kouraklis G. (2012). Papillary thyroid microcarcinoma: clinicopathological characteristics and implications for treatment in 276 patients. Eur J Clin Invest.

[8] Roti E., degli Uberti E.C., Bondanelli M., Braverman L.E. (2008). Thyroid papillary microcarcinoma: a descriptive and metaanalysis study. Eur J Endocrinol.

[9] Niemeier L.A., Kuffner Akatsu H., Song C., Carty S.E., Hodak S.P., Yip L. (2012). A combined molecular-pathologic score improves risk stratification of thyroid papillary microcarcinoma. Cancer.

[10] Webb R.C., Howard R.S., Stojadinovic A., Gaitonde D.Y., Wallace M.K., Ahmed J. (2012). The utility of serum thyroglobulin measurement at the time of remnant ablation for predicting disease-free status in patients with differentiated thyroid cancer: a meta-analysis involving 3947 patients. J Clin Endocrinol Metab.

[11] DeLellis R.A., Lloyd R.V., Heitz P.U., Eng C. (2004).

[12] Pitoia F., Bueno F., Urciuoli C., Abelleira E., Cross G., Tuttle R.M. (2013). Outcomes of patients with differentiated thyroid cancer risk-stratified according to the American thyroid association and Latin American thyroid society risk of recurrence classification systems. Thyroid.

[13] Edge S.B., Byrd D.R., Compton C.C., Fritz A.G., Greene F., Trotti A. (2010). Thyroid.

[14] Tuttle R.M., Tala H., Shah J., Leboeuf R., Ghossein R., Gonen M. (2010). Estimating risk of recurrence in differentiated thyroid cancer after total thyroidectomy and radioactive iodine remnant ablation: using response to therapy variables to modify the initial risk estimates predicted by the new American Thyroid Association staging system. Thyroid.

[15] Padovani R.P., Robenshtok E., Brokhin M., Tuttle R.M. (2012). Even without additional therapy, serum thyroglobulin concentrations often decline for years after total thyroidectomy and radioactive remnant ablation in patients with differentiated thyroid cancer. Thyroid.

[16] Hussain S.Z., Zaman M., Malik S., Ram N., Asghar A., Rabbani U. (2014). Preablation stimulated thyroglobulin/TSH ratio as a predictor of successful I^131^ remnant ablation in patients with differentiated thyroid cancer following total thyroidectomy. J Thyroid Res.

[17] Trevizam P.G., Tagliarini J.V., Castilho E.C., de Alencar Marques M., Kiy Y., da Silva Mazeto G.M. (2017). Thyroglobulin levels and thyroglobulin/thyrotropin ratio could predict the success of the ablative/therapeutic ^131^I in the differentiated thyroid cancers. Endocr Res.

[18] Kuo S.F., Chao T.C., Chang H.Y., Hsueh C., Yang C.H., Lin J.D. (2011). Prognostic evaluation of patients with multicentric papillary thyroid microcarcinoma. J Formos Med Assoc.

[19] Usluogullari C.A., Onal E.D., Ozdemir E., Ucler R., Kiyak G., Ersoy P.E. (2015). A retrospective analysis of prognostic factors predictive of lymph-node metastasis and recurrence in thyroid papillary microcarcinoma. Minerva Endocrinol.

[20] Pyo J.S., Sohn J.H., Kang G. (2016). Detection of tumor multifocality is important for prediction of tumor recurrence in papillary thyroid microcarcinoma: a retrospective study and meta-analysis. J Pathol Transl Med.

[21] Macedo F.I., Mittal V.K. (2015). Total thyroidectomy versus lobectomy as initial operation for small unilateral papillary thyroid carcinoma: a meta-analysis. Surg Oncol.

[22] Kaliszewski K., Wojtczak B., Strutyńska-Karpińska M., Łukieńczuk T., Forkasiewicz Z., Domosławski P. (2016). Incidental and non-incidental thyroid microcarcinoma. Oncol Lett.

[23] Girardi F.M., Barra M.B., Zettler C.G. (2015). Analysis of pattern of occurrence of thyroid carcinoma between 2001 and 2010. Braz J Otorhinolaryngol.

